# Chimeric Virus-like Particles Formed by the Coat Proteins of Single-Stranded RNA Phages Beihai32 and PQ465, Simultaneously Displaying the M2e Peptide and the Stalk HA Peptide from Influenza a Virus, Elicit Humoral and T-Cell Immune Responses in Mice

**DOI:** 10.3390/vaccines13111117

**Published:** 2025-10-30

**Authors:** Egor A. Vasyagin, Anna A. Zykova, Elena A. Blokhina, Olga O. Ozhereleva, Liudmila A. Stepanova, Marina A. Shuklina, Sergey A. Klotchenko, Eugenia S. Mardanova, Nikolai V. Ravin

**Affiliations:** 1 Institute of Bioengineering, Research Center of Biotechnology of the Russian Academy of Sciences, 119071 Moscow, Russia; 2 Smorodintsev Research Institute of Influenza, Russian Ministry of Health, 197376 St. Petersburg, Russia

**Keywords:** virus-like particle, vaccine, ssRNA bacteriophage, M2e peptide, hemagglutinin, influenza A virus, T-cells

## Abstract

**Background:** The extracellular domain of the M2 protein (M2e) and the conserved region of the second subunit of the hemagglutinin (HA2, 76–130 а.а.) of the influenza A virus, could be used to develop broad-spectrum influenza vaccines. However, these antigens have low immunogenicity and require the use of special carriers to enhance it. Virus-like particles (VLPs) formed from viral capsid proteins are among the most effective carriers. **Methods:** In this work, we obtained and characterized VLPs based on capsid proteins (CPs) of single-stranded RNA bacteriophages Beihai32 and PQ465, simultaneously displaying M2e and HA2 peptides. **Results:** Fusion proteins expressed in *Escherichia coli* formed spherical VLPs of about 30 nm in size. Subcutaneous immunization of mice with chimeric VLPs elicited a robust humoral immune response against M2e and the whole influenza A virus, and promoted the formation of cytokine-secreting antigen-specific CD4^+^ and CD8^+^ effector memory T cells. **Conclusions:** VLPs based on CPs of phages Beihai32 and PQ465 carrying conserved peptides M2e and HA2 of the influenza A virus can be used for the development of universal influenza vaccines.

## 1. Introduction

Seasonal influenza viruses, due to their continuous evolution and high level of infectiousness, pose a significant threat to public health worldwide every year. According to the World Health Organization (WHO), infection leads to 3 to 5 million cases of severe illness and 290,000 to 650,000 deaths annually [1,2,3]. The high prevalence of influenza highlights the importance of measures to control and prevent influenza virus infections. The most effective means of preventing influenza is vaccination. A distinctive feature of influenza viruses is the ability to undergo rapid antigenic change due to the accumulation of mutations in the antibody-binding sites of the hemagglutinin (HA) and neuraminidase (NA) surface proteins, resulting in the absence of binding of some antibodies produced as a result of previous vaccinations or infections.

Every year, seasonal influenza viruses pose a serious threat to public health around the world because of their high level of contagiousness and ongoing evolution. The World Health Organization (WHO) estimates that each year, influenza infections cause between 290,000 and 650,000 deaths and 3 to 5 million episodes of severe illness [1,2,3]. Vaccination is the most effective way to prevent influenza. However, because of the accumulation of mutations in the antibody-binding sites of the hemagglutinin (HA) and neuraminidase (NA) surface proteins, influenza A viruses undergo rapid antigenic change. Therefore, antibodies produced as a result of previous vaccinations or infections may not be active against the new strain of the virus [4]. This antigenic drift requires regular updating of vaccines. Continuous surveillance of circulating influenza viruses is crucial for the selection of relevant influenza virus strains and timely vaccine production. [5,6].

Effective control of the spread of influenza A virus could potentially be achieved by developing a broad-spectrum vaccine based on conserved viral antigens. A number of studies have shown that vaccine candidates, -based on conserved influenza virus proteins can ensure a protective immune response against a wide range of influenza A subtypes [7,8]. Modern universal influenza vaccine development increasingly focuses on the construction of fusion proteins that incorporate multiple conserved viral antigens. Numerous studies have confirmed both the feasibility and efficacy of this strategy [9,10,11,12,13]. Highly conserved antigens, the surface-exposed extracellular domain of the M2 protein (M2e) and the stalk region of hemagglutinin (HA2), served as effective targets for developing broadly protective influenza A vaccine candidates [14,15,16,17,18,19,20]. However, neither conventional vaccination nor natural influenza infection induces substantial levels of anti-HA2 or anti-M2e antibodies [21,22,23], primarily because these conserved epitopes are overshadowed by the immunodominant HA1 region. The use of M2e and HA2 antigens alone is limited by their low immunogenicity. However, linking M2e and/or HA2 to highly immunogenic protein carriers can overcome this limitation [18,24].

Virus-like particles (VLPs), nanoscale structures formed from viral capsid proteins through self-assembly, are widely used in vaccine development. Several VLP-based vaccines have already been approved for use, namely against human papillomavirus, hepatitis B virus, and hepatitis E virus, while many others are in different stages of clinical trials [25]. VLP-based antigen display serves as a potent method to boost immune responses to foreign antigens [26,27,28,29,30]. VLPs mimic the viral structure and efficiently interact with dendritic cells (DCs). This interaction facilitates antigen presentation via both MHC class I and II pathways, prompting DC migration to lymph nodes. Here, they stimulate Th1 and Th2 responses, inducing robust humoral and cellular immunity which makes VLPs an attractive vaccine platform [31]. Several studies have also demonstrated this approach’s effectiveness for influenza A epitope presentation [32].

Bacteriophage capsid proteins (CPs) are a promising platform for antigen display because they can be readily produced in large quantities in bacterial systems, such as *Escherichia coli*. Bacteriophages of the *Fiersviridae* family (formerly *Leviviridae*) are particularly promising as a VLP platform [33]. Their spherical virions (approximately 30 nm in diameter) consist of approximately 180 copies of the major capsid protein, and contain a 3–4 kb linear ssRNA(+) genome [34]. CPs of these bacteriophages, expressed in bacteria, can self-assemble to form VLPs whose structure is similar to that of the virion. Furthermore, expression of a hybrid CP genetically fused with a heterologous peptide allows for the production of chimeric VLPs displaying this peptide on their surface. Such chimeric VLPs carrying antigens were previously obtained using CPs of bacteriophages AP205, MS2, PP7, Qβ, and P22 [25].

However, the inclusion of foreign peptides in the chimeric CP can alter its spatial structure and interfere with VLP assembly. Such effects are more pronounced for large inclusions (tens to hundreds of a.a.). The largest insertions into the CPs of the aforementioned phages, which did not interfere with the assembly of the chimeric VLPs, were no larger than 124 a.a. [35,36,37].

In our previous work, we constructed VLPs based on CPs of single-stranded RNA phages Beihai32 and PQ465 bearing four copies of the M2e peptide (92 a.a. in total). These chimeric VLPs induced high levels of M2e-specific IgG antibodies in the serum of immunized mice and protected them from lethal influenza A virus challenge [38]. Recently, using the example of green fluorescent protein (238 a.a.), we showed that the capsid proteins of these phages can include longer peptides that are displayed on the surface of chimeric VLPs [39].

Taking advantage of this opportunity, in the current study, we aimed to improve the efficacy of the candidate vaccine by incorporating both four copies of the M2e peptide and the HA stalk domain (HA2, a.a. 76–130) into VLPs based on CPs of phages Beihai32 and PQ465. We obtained chimeric VLPs and showed that they could induce humoral and T-cell immune responses in mice.

## 2. Materials and Methods

### 2.1. Design of Fusion Proteins and Construction of Expression Vectors

Plasmid pETM10 [40] was used for the expression of recombinant proteins. The M2e peptide sequence matched the consensus M2e sequence of human influenza A viruses [41], with two cysteine residues replaced by serines to prevent the formation of disulfide bonds that lead to protein aggregation (SLLTEVETPIRNEWGSRSNDSSD; cysteine-to-serine substitutions are underlined) [38]. Additionally, a fragment corresponding to the highly conserved region of the second subunit of hemagglutinin (HA2, a.a. 76–130) representative of phylogenetic group 2 influenza A viruses (HA2-2, RIQDLEKYVEDTKIDLWSYNAELLVALENQHTIDLTDSEMNKLFEKTRRQLRENA) [19] was incorporated.

The DNA fragment encoding the HA2 sequence was obtained by PCR amplification using primers (Ecl136II-HA2 F: ATGAGCTCCGTATTCAGGATCTGGA, HA2-EcoRV R: TAGATATCCGCATTTCACGCAGCT), that introduced restriction site *Ecl*136II at the N-terminus and *EcoR*V at the C-terminus of the HA2 fragment. The recombinant vector pQE30_Flg_HA2–2_4M2e [19] was used as a template. The PCR fragment was cloned at the *Ecl*136II site into the previously obtained recombinant vector pETM-10 Beihai32-19S-4M2eh [38], which encodes hexahistidine tags both at the N- and C- ends of the insert. Thus, expression vector pETM-10 Beihai32-19S-HA2-4M2eh, which encodes CP of Behai32 with a HA2 fragment and four M2e copies separated from CP by a flexible glycine-serine linker and containing hexahistidine tags at both the N- and C-termini, was obtained.

To replace the Beihai32 CP sequence with the PQ465 CP gene, *BamH*I/*Pvu*I DNA fragment was excised from the previously constructed plasmid pETM10_PQ465-19S-4M2eh [38] and used for cloning at the same restriction sites of pETM-10 Beihai32-19S-HA2-4M2eh. As a result, a new expression vector, pETM-10 PQ465-19S-HA2-4M2eh, was constructed.

3D structures of recombinant proteins were generated using AlphaFold v2.3.1 [42] and visualized with the SWISS-MODEL server [43].

### 2.2. Expression of Recombinant Proteins

The *E. coli* strain BL21 (DE3) was used as a host for the expression of recombinant proteins. The culture was grown in LB medium at 37 °C with shaking at 130 rpm until the middle of the logarithmic growth phase (OD_600_~0.5). Expression of the target protein was induced by the addition of 0.5 mM isopropyl β-D-1-thiogalactopyranoside (IPTG). Culture growth was continued overnight at 20 °C. Cells were then collected by centrifugation, resuspended in 10 mM PBS (pH 7.2) with lysozyme (1 mg/mL) and incubated for 15 min at room temperature. After freezing at −20 °C overnight and thawing on ice, cells were lysed by sonication using a SONOPULS HD 2200 ultrasonic homogenizer (Bandelin SONOPULS, Bandelin Electronic GmbH, Berlin, Germany). The lysate was centrifuged at 14,000× *g*, and the supernatant was collected and used for purification of the recombinant proteins.

### 2.3. Purification of VLPs

For the purification of the recombinant proteins Beihai32-19S-HA2-4M2eh and PQ465-19S-HA2-4M2eh, saturated ammonium sulfate was added to the cell lysates at a ratio of 1:2 (saturated ammonium sulfate:cell lysate, *v*/*v*) and the mixtures were incubated overnight at 4 °C. After incubation, the samples were centrifuged at 14,000× *g*, and the supernatants were discarded. The resulting pellets were resuspended in 10 mM PBS, followed by a second centrifugation step at 14,000× *g*. The supernatants containing the target proteins were transferred to clean tubes.

The PQ465-19S-HA2-4M2eh protein was further subjected to a second round of precipitation using saturated ammonium sulfate at a ratio of 1:3 (*v*/*v*) with overnight incubation and subsequent centrifugation, followed by dialysis against 10 mM PBS.

The Beihai32-19S-HA2-4M2eh protein was further purified using metal-affinity chromatography on Ni-NTA agarose (Qiagen, Hilden, Germany). Protein binding to the sorbent was performed for 1 h in 10 mM PBS containing 1 M NaCl. Unbound proteins were removed by washing with the same buffer supplemented with 16 mM imidazole. The target protein was eluted from the sorbent with 10 mM PBS containing 500 mM imidazole. The eluted protein was dialyzed against 10 mM PBS and stored at −20 °C.

The proteins remained stable at −20 °C for at least one year, as determined by SDS-PAGE. After freeze–thaw, the VLPs remained intact, as confirmed by electron microscopy.

### 2.4. Structural Analysis of VLPs

The formation of VLPs and their structure were analyzed using transmission electron microscopy (TEM) and dynamic light scattering. For TEM analysis the samples were loaded onto carbon-coated copper grids and stained with 1% (*w*/*v*) uranyl acetate. The grids were then analyzed using a JEM 1400 transmission electron microscope (JEOL, Tokyo, Japan). Dynamic light scattering experiments were performed using a Zetasizer NanoS90 analyzer (Malvern Panalytical, Malvern, UK) with 12 mm square polystyrene cuvettes.

### 2.5. Immunization of Mice

The study used female *BALB*/*c* (haplotype H-2d) and *C57Bl6* (haplotype H-2b) mice weighing 17–19 g. Mice were randomly assigned to groups, with no animals excluded.

To evaluate the immunogenicity of the chimeric VLPs, *BALB*/*c* mice (5 per group) were immunized with purified Beihai32-19S-HA2-4M2eh and PQ465-19S-HA2-4M2eh particles. The mice were immunized subcutaneously three times, with a two-week interval, at a dose of 50 μg per mouse (50 μL) without adjuvants. Mice in the control groups (5 per group) were similarly administered with VLPs formed by empty capsid proteins of Behai32 and PQ465, obtained as described in a previous study [38], and with PBS.

In the second experiment, aimed to characterize the T-cell response, *C57Bl*/*6* mice (5 per group) were immunized with Beihai32-19S-HA2-4M2eh and PQ465-19S-HA2-4M2eh particles. The mice were immunized subcutaneously three times, with a two-week interval, at a dose of 50 μg per mouse (50 μL) without adjuvants. The control group (5 mice) was similarly injected with PBS.

No side effects of vaccination, such as death of animals, weight loss or loss of appetite, were observed, indicating the vaccine was well-tolerated in the experimental setting.

### 2.6. Analysis of Antibody Titers by ELISA

Sera and bronchoalveolar lavage (BAL) samples from five *BALB*/*c* mice in each group were taken on the fourteenth day following the third immunization in order to measure antibody titers. The enzyme-linked immunosorbent assay (ELISA) was used to measure the antibody titers. 96-well microtiter plates (Greiner Bio-One GmbH, Kremsmünster, Austria) were coated with 5 µg/mL of the synthetic peptide G37, corresponding to the consensus sequence of M2e from human influenza A strains (SLLTEVETPIRNEW GCRCNDSSD) or with the influenza virus A/Aichi/2/68 (H3N2) at a concentration of 1 µg/mL. Fetal bovine serum (5%) was used to block the plates. Two-fold serial dilutions of each serum, starting from 1/400, were added to the wells. Goat anti-mouse IgG, IgG1, and IgG2a coupled with horseradish peroxidase (Abcam, Cambridge, UK) were used as secondary antibodies. The detection substrate was tetramethylbenzidine (Biolegend, San Diego, CA, USA). H_2_SO_4_ was used to halt the reaction, and a microplate reader was used to determine the optical density (OD) at 450 nm. The highest serum dilution at which the OD was at least twice the average value of the blank experiment was taken as the antibody titer.

### 2.7. Isolation of Cells from Spleens to Analyze the T-Cellular Immune Response

Two weeks after the last immunization, five C57Bl6 mice from the experimental and control groups were euthanized in a CO_2_ chamber. The spleens of mice were aseptically removed and homogenized using a Medimachine (BD Biosciences, San Jose, CA, USA) with Medicons (BD Biosciences, USA) and 70-µm Syringe Filcons (BD Biosciences, USA). The cell solution was washed with PBS containing 2% Fetal Bovine Serum (FBS) and centrifuged at 500× *g* for 5 min at 10 °C. 4 mL of RBC Lysis Buffer (BioLegend, USA) was used to lyse the erythrocytes for 3–5 min. 10 mL of PBS + 2% FBS was then added, and the cells were centrifuged at 500× *g* for 5 min at 10 °C. After removing the supernatant, 4 mL of full RPMI 1640 media containing 100 µg/mL streptomycin and 100 µg/mL penicillin was added. The cells were resuspended and adjusted to a final concentration of 10^7^ cells/mL. A CytoFLEX flow cytometer (Beckman Coulter, Brea, CA, USA) was used to count the cells.

### 2.8. Determination of Antigen-Specific Effector Memory T-Cells Using Multiparametric Flow Cytometry

The formation of antigen-specific CD4^+^ and CD8^+^ effector memory T-lymphocytes (Tem) producing cytokines in the spleen of immunized mice was evaluated by multiparametric flow cytometry according to the BD Pharmingen^TM^ protocol.

Cell activation was performed in flat-bottom culture plates. Cells were activated with the synthetic peptide G-37 (M2eh) (5 µg) for 6 h or with the A/Aichi/2/68 (H3N2) virus (1 µg) for 24 h, in the presence of brefeldin A (1 µg/mL) (BD Biosciences, USA) and CD107a-PE/Dazzle 594 antibodies. After activation, cells were transferred to conical-bottom plates to reduce cell loss.

The Fc receptors were blocked using CD16/CD32 antibodies (TruStain FcX, BioLegend, USA), followed by incubation with Zombie Aqua (Zombie Aqua Fixable Viability Kit, BioLegend, USA) to distinguish viable cells. The cells were stained with fluorescently labeled antibodies: CD3a-FITC, CD4-PerCP/Cyanine5.5, CD8-PE/Cyanine7, CD62L-APC/Cyanine7, and CD44-Brilliant Violet 510^TM^ (BioLegend, USA) at 2–8 °C for 30 min. Subsequently, the cells were permeabilized according to the Cytofix/Cytoperm Plus test system protocol (BD Biosciences, USA) and stained with TNF-α-Brilliant Violet 421^TM^, IFN-γ-FITC, and IL-2-PE (BioLegend, USA). Data collection (100,000 viable CD3+ lymphocytes) was acquired using a Cytoflex flow cytometer (Beckman Coulter, Brea, CA, USA).

### 2.9. Analysis of Antigenic Properties of VLPs by ELISA

ELISA plates were coated with serial two-fold dilutions of VLPs of Beihai32, Beihai32-19S-HA2-4M2eh, PQ465, and PQ465-19S-HA2-4M2eh in sodium bicarbonate buffer (pH 8.5) overnight at 4 °C. After washing with PBST (PBS with 0.05% Tween), the plates were treated with a blocking buffer (0.2% (*w*/*v*) BSA in PBS) for one hour at 37 °C. Then the plates were washed with PBS and probed with mouse polyclonal antibodies against M2e, HA2, Beifhai32, and PQ465 for 30 min at 37 °C. After that, PBST was used to wash the plates five times. Anti-mouse antibodies labeled with peroxidase were then added to the wells. After 30 min incubation, the wells were rinsed five times with PBST. Horseradish peroxidase activity was detected using tetramethylbenzidine (Vector-BEST, Novosibirsk, Russia). HCI (0.5 N) was added to halt the reaction, and a microplate spectrophotometer was used to determine the optical density at 450 nm.

### 2.10. Statistical Analysis

Statistical analyses were carried out using GraphPad Prism v.10.4. Group comparisons of antibody titers obtained by ELISA; T-cell response data were assessed using ANOVA followed by Tukey’s multiple comparisons test. A threshold of *p* < 0.05 was used to determine statistical significance. Flow cytometry data was analyzed using Kaluza 2.2 (Beckman Coulter). T-cell response data was visualized as histograms and Tukey graphs. Statistical significance was assessed via ANOVA in GraphPad Prism v.10.4, with *p* < 0.05 considered significant.

### 2.11. T-Cell and B-Cell Epitopes Search In Silico

We predicted MHCII and MHCI binding predicted using the IEDB NetMHCIIpan v. 4.1 tool [44] and IEDB NetMHCpan v. 4.1 [45] analysis resource tool, respectively. B-cell epitope predictions were made using the Bepipred Linear Epitope Prediction tool [46].

### 2.12. Ethics Statement

The study was carried out according to the recommendation of the Board of the Eurasian Economic Commission “On the Guidelines for working with laboratory (experimental) animals when conducting preclinical (non-clinical) studies” (14 November 2023, No 33). The experiments were approved by the Bioethics Committee on the Use of Animals of the Smorodintsev Research Institute of Influenza (Permit ID 24 dated 29 August 2024). All possible efforts were made to minimize the suffering of the animals.

## 3. Results

### 3.1. Design, Expression and Purification of Recombinant Proteins

**Figure 1:**
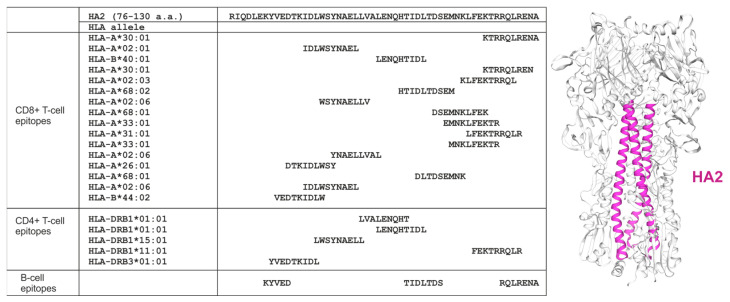
Potential CD8^+^ T-cell, CD4^+^ T-cell and B-cell epitopes in the HA2 fragment.

**Figure 2:**
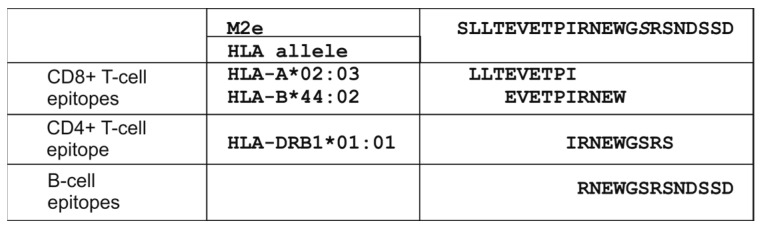
Potential CD8^+^ T-cell, CD4^+^ T-cell and B-cell epitopes in the M2e peptide.

The study targeted two conserved influenza A antigens: the M2e peptide, consensus for human strains (M2eh), and the HA2 fragment (a.a. 76–130), consensus for influenza viruses of the second phylogenetic group (H3, H4, H7, H10, H14, H15) [18]. This HA segment comprised the major α-helical domain of HA2 and demonstrated partial surface accessibility (Figure 1). CD8^+^ and CD4^+^ Т-cell epitopes of various HLA-alleles as well as B-cell epitopes were predicted in M2e (Figure 2) and the HA2 fragment (Figure 1).

**Figure 3:**
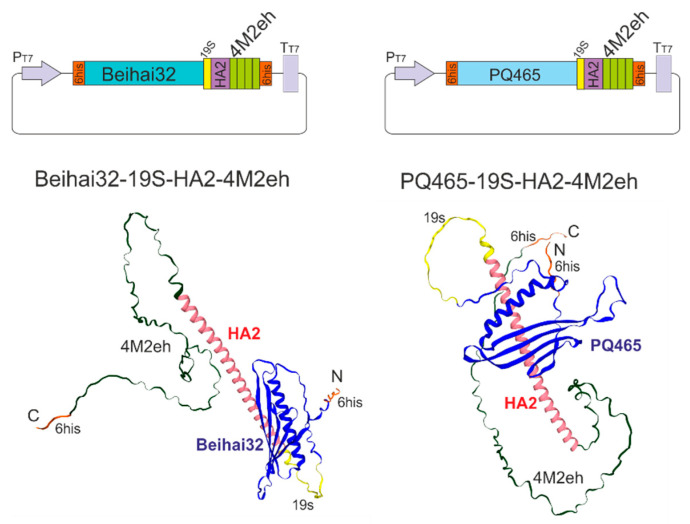
Scheme of the expression vectors. P_T7_, bacteriophage T7 promoter for *E. coli* RNA polymerase, with embedded *lac* operator; T_T7_, bacteriophage T7 terminator; HA2, the HA fragment (76–130 a.a.); 4M2eh, four tandem copies of the M2e peptide; Beihai32, CP of bacteriophage Beihai32; PQ465, CP of bacteriophage PQ465; 19S, flexible glycine/serine-rich linker; 6his, sequence that encodes 6-histidine tag. Predicted 3D structures of monomeric proteins are shown below.

The recombinant proteins Beihai32-19S-HA2-4M2eh and PQ465-19S-HA2-4M2eh contained HA2 along with four tandem copies of the M2e peptide. To prevent protein aggregation, cysteine residues at positions 17 and 19 of M2e were replaced with serines, a modification previously shown not to affect the immunogenicity of M2e [47]. These peptides were fused to the C-terminus of capsid proteins of phages PQ465 and Beihai32 via a glycine-serine-rich 19S linker, which was used to facilitate proper folding and ensure effective epitope display on the surface of the chimeric particles. Additionally, both recombinant proteins were equipped with N- and C-terminal hexahistidine tags to enable their purification by metal-affinity chromatography (Figure 3). The amino acid sequences of the recombinant proteins are shown in Supplementary Figure S1.

**Figure 4:**
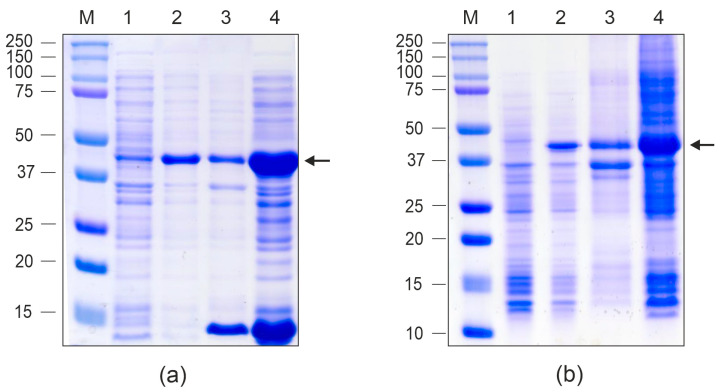
Expression of the recombinant proteins in *E. coli*. The proteins were analyzed using SDS-PAGE. M, molecular weight marker (sizes are shown in kDa). Proteins isolated from *E. coli* cultures before induction, total protein (lane 1), upon induction, total protein (lane 2), upon induction, insoluble fraction (lane 3), upon induction, soluble fraction (lane 4); (**a**) Beihai32-19S-HA2-4M2eh; (**b**) PQ465-19S-HA2-4M2eh. The positions of the target proteins are shown with arrows.

The designed proteins were expressed in *E. coli* using vectors pETM-10 Beihai32-19S-HA2-4M2eh and pETM-10 PQ465-19S-HA2-4M2eh (Figure 3). Protein expression was induced at 20 °C, resulting in high-level synthesis of both recombinant proteins, which were predominantly detected in the soluble fraction of the cell lysate (Figure 4).

**Figure 5:**
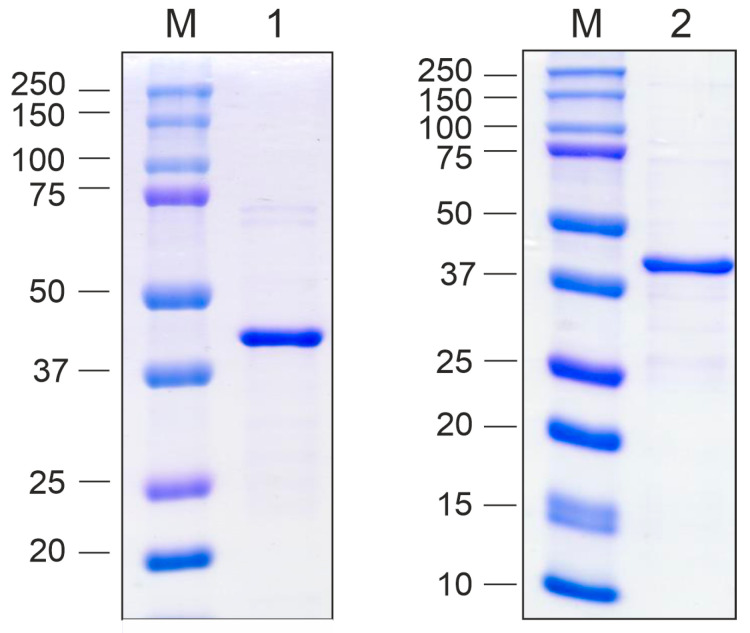
Purification of the recombinant proteins produced in *E. coli*. The proteins were analyzed using SDS-PAGE. M, molecular weight marker (sizes are shown in kDa). Purified proteins: Beihai32-19S-HA2-4M2eh (lane 1); PQ465-19S-HA2-4M2eh (lane 2).

At the initial step of purification, VLPs formed by the recombinant proteins were subjected to a two-step precipitation using a saturated ammonium sulfate solution. Subsequently, Beihai32-19S-HA2-4M2eh was further purified using Ni-NTA resin. In contrast, PQ465-19S-HA2-4M2eh exhibited poor affinity for the resin and was therefore subjected to an additional round of ammonium sulfate precipitation (Figure 5).

### 3.2. Assembly of Irus-like Particles from Chimeric Coat Proteins Fused with Influenza a Virus Antigens

**Figure 6:**
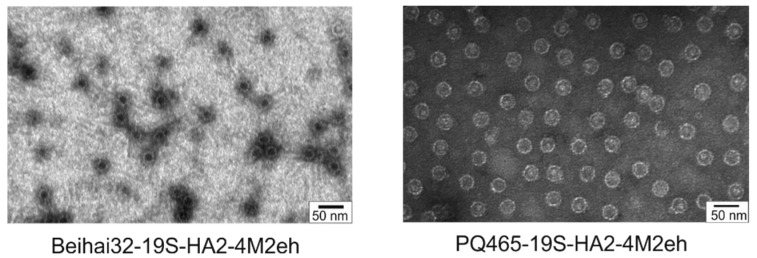
Analysis of the chimeric VLPs by transmission electron microscopy.

For subsequent analysis of VLPs assembly, the purified proteins were dialyzed against PBS and examined using transmission electron microscopy. The analysis revealed that both Beihai32-19S-HA2-4M2eh and PQ465-19S-HA2-4M2eh proteins self-assembled into VLPs approximately 30 nm in diameter (Figure 6).

The particles were further analyzed using dynamic light scattering. The sizes of Beihai32-19S-HA2-4M2eh and PQ465-19S-HA2-4M2eh VLPs estimated by this method were 61.4 ± 7.4 nm and 69.6 ± 8.4 nm, respectively. Minor fractions of larger aggregates of approximately 200 nm in size were also observed (Supplementary Figure S2).

**Figure 7:**
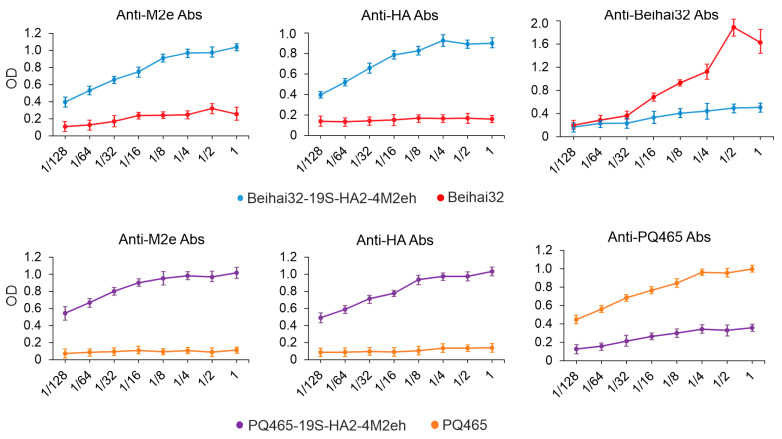
Antigenic characteristics of VLPs. Two-fold dilutions of VLPs formed by Beihai32, Beihai32-19S-HA2-4M2eh, PQ465, and PQ465-19S-HA2-4M2eh proteins were loaded on ELISA plates and probed with antibodies against M2e, HA2, PQ465, or Beihai32. After incubation with the secondary antibodies labeled with peroxidase, peroxidase activity was detected using tetramethylbenzidine, and the optical density at 450 nm was recorded. Mean values of measured OD and error bars representing standard deviation (measurements from three wells) are shown.

The three-dimensional positioning of antigenic insertions within chimeric VLPs critically affects presentation efficiency by controlling epitope surface exposure. Experimentally, the antigenic properties of the obtained VLPs were assessed using ELISA with polyclonal antibodies specific to M2e and HA2 (Figure 7). VLPs formed by Beihai32-19S-HA2-4M2eh and PQ465-19S-HA2-4M2eh proteins were efficiently recognized by both M2e and HA2-specifc antibodies, suggesting that insertions comprising M2e and HA2 were exposed on the surface of VLPs. The data obtained also indicated the partial masking of the carrier CP by the attached antigens.

### 3.3. Analysis of the Humoral Immune Response Induced by Chimeric VLPs

**Figure 8:**
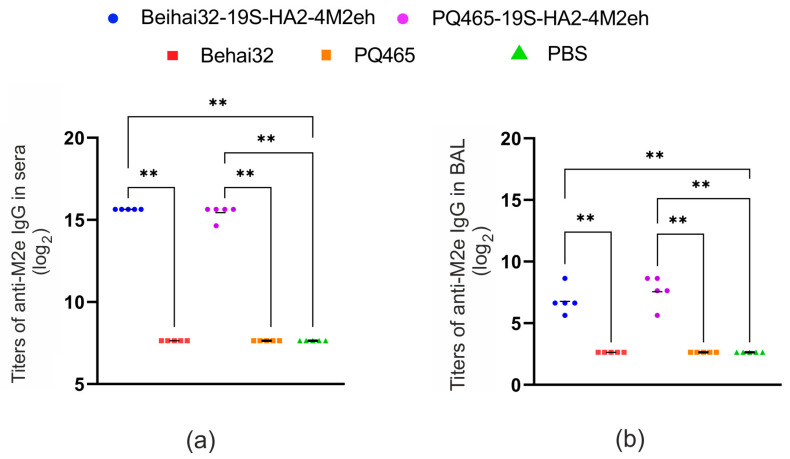
Immunogenicity of chimeric VLPs in mice: induction of anti-M2e antibodies. ELISA was used to determine the titers of anti-M2e IgG in sera (**a**) and BAL (**b**) of mice (5 per group). Geometric mean titers and endpoint titers observed in individual mice are shown. The asterisks (** *p* < 0.0001) indicate the statistical significance of the differences between groups. Exact *p*-values are shown in Supplementary Table S1.

Beihai32-19S-HA2-4M2eh and PQ465-19S-HA2-4M2eh VLPs along with control Beihai32 and PQ465 particles were subcutaneously administered to *BALB*/*c* mice in order to assess immunogenicity. Serum and BAL samples were obtained after the third vaccination, and IgG antibodies specific to the M2e peptide and influenza A/Aichi/2/68 (H3N2) virus were detected by ELISA (Figure 8).

For both Beihai32-19S-HA2-4M2eh and PQ465-19S-HA2-4M2eh, high titers of M2e-specific antibodies were detected in the sera of immunized mice, with levels significantly exceeding those in the control groups immunized with PBS or empty capsid proteins (Figure 8). These results are consistent with our previous findings [38], and showed that incorporation of the HA2 fragment did not compromise the immunogenicity of the chimeric VLPs regarding M2e. Anti-M2e IgG titers in the BAL fluids were also significantly higher than those in the control groups, although the overall levels were lower compared to those observed in serum (Figure 8).

**Figure 9:**
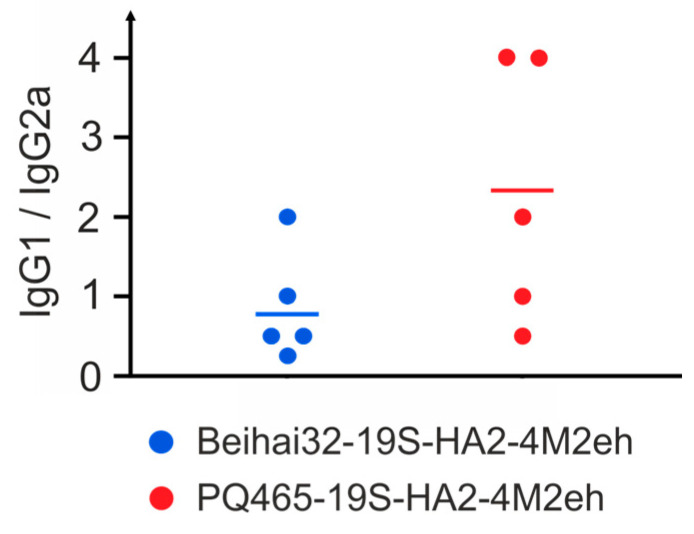
Ratio of IgG1 and IgG2a antibodies against M2e in sera of mice (5 per group) immunized with chimeric VLPs.

In addition to measuring total IgG antibody levels, we also analyzed the levels of IgG1 and IgG2a subclasses, which serve as markers of Th2- and Th1-type immune responses, respectively. A balanced Th1/Th2 immune response was observed in mice immunized with Beihai32-19S-HA2-4M2eh, while PQ465-19S-HA2-4M2eh particles induced stronger IgG1 response, suggesting a shift toward Th2 polarization (Figure 9).

**Figure 10:**
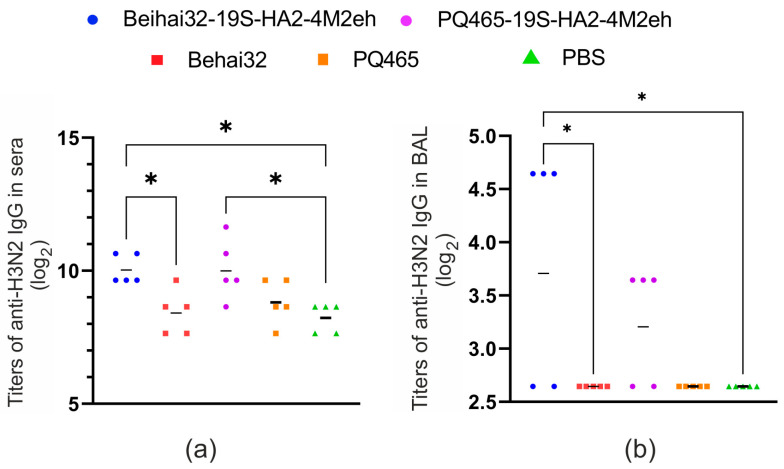
Immunogenicity of chimeric VLPs in mice: induction of antibodies against influenza virus A/Aichi/2/68 (H3N2). ELISA was used to determine the titers of anti-H3N2 IgG in sera (**a**) and BAL (**b**) of mice (5 per group). Geometric mean titers and endpoint titers observed in individual mice are shown. The asterisks (* *p* < 0.05) on top of the lines indicate the statistical significance of the differences between groups. Exact *p*-values are shown in Supplementary Table S1.

Because the chimeric VLPs displayed the HA2 peptide, we also examined the production of antibodies binding to influenza virus A/Aichi/2/68 (H3N2) in sera and BAL samples. Immunization with Beihai32-19S-HA2-4M2eh induced virus-specific IgG antibodies detectable in both serum and BAL, with titers significantly higher than in the control groups (Figure 10). In the case of PQ465-19S-HA2-4M2eh, virus-specific antibody formation was observed only in the serum, with a significant difference compared with the PBS control group (Figure 10).

### 3.4. Chimeric VLPs Induced Antigen-Specific T-Cell Response in Spleen

To characterize the T-cell response, lymphocytes were isolated from the spleens of immunized and control *C57Bl*/*6* mice two weeks after the last immunization. They were stimulated with the M2e peptide or the A/Aichi/2/68 (H3N2) virus. The T-cell response was characterized by evaluating the capacity of M2e-specific and virus-specific CD4^+^ and CD8^+^ effector memory T cells (Tem) to produce cytokines (IL-2, TNF-α, and IFN-γ) by intracellular cytokine staining assay. The gating strategy for cytokine-secreting antigen-specific CD4^+^ and CD8^+^ Tem cells and representative plots are shown in Supplementary Figures S3 and S4.

**Figure 11:**
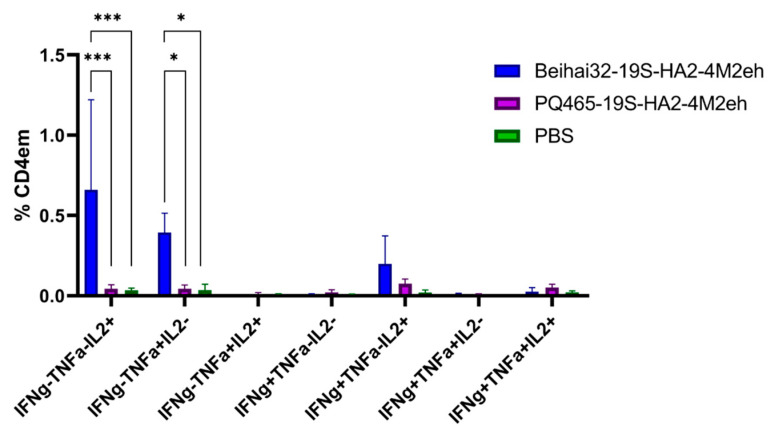
Cytokine profile of M2e-specific CD4^+^ Tem in the spleens of C57Bl6 mice (5 per group) after subcutaneous immunization. The *p* values between groups are indicated (* *p* < 0.05; *** *p* < 0.001). Exact *p*-values are shown in Supplementary Table S1.

Subcutaneous immunization with Beihai32-19S-HA2-4M2eh particles induced the formation of M2e-specific CD4^+^ Tem response (Figure 11). The M2e-specific CD4^+^ Tem population included IL-2 (0.66%) and TNF-α (0.40%) mono-producers, which amounts significantly differed from the groups of mice immunized with PQ465-19S-HA2-4M2eh and PBS (Figure 11). The amounts of M2e-specific polyfunctional CD4^+^ Tem cells co-expressing two or three cytokines did not differ significantly between the experimental and control groups. Immunization with PQ465-19S-HA2-4M2eh particles did not lead to the formation of M2e-specific CD4^+^ Tem response.

**Figure 12:**
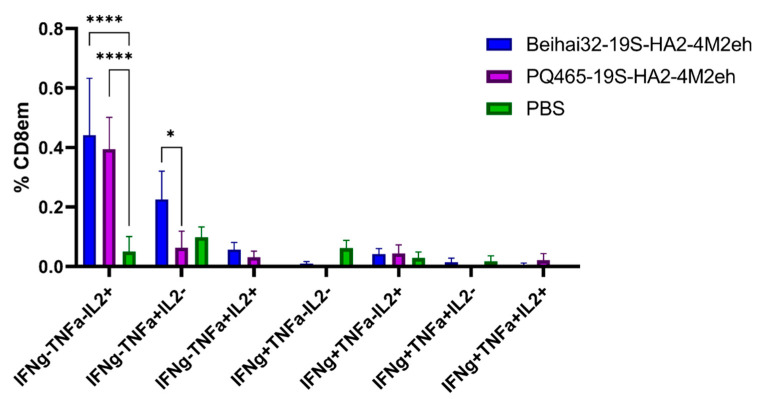
Cytokine profile of M2e-specific CD8^+^ Tem in the spleens of C57B/6 mice (5 per group) after subcutaneous immunization. The data is presented in the form of Tukey plots. The *p* values between groups are indicated (* *p* < 0.05; **** *p* < 0.001). Exact *p*-values are shown in Supplementary Table S1.

Subcutaneous immunization of *C57Bl*/*6* mice with Beihai32-19S-HA2-4M2eh and PQ465-19S-HA2-4M2eh particles significantly increased the number of M2e-specific CD8^+^ Tem detected in the spleen compared with control mice (PBS). Immunization stimulated the formation of M2e-specific CD8^+^ Tem, producing IL-2 (0.44% and 0.39%, respectively), with a significant difference from the control (*p* < 0.0001). Additionally, Beihai32-19S-HA2-4M2eh particles induced the formation of CD8^+^ Tem mono-producers of TNF-α (0.23%) (Figure 12). The amounts of M2e-specific polyfunctional CD8^+^ Tem cells co-expressing two or three cytokines did not differ significantly between the experimental and control groups.

**Figure 13:**
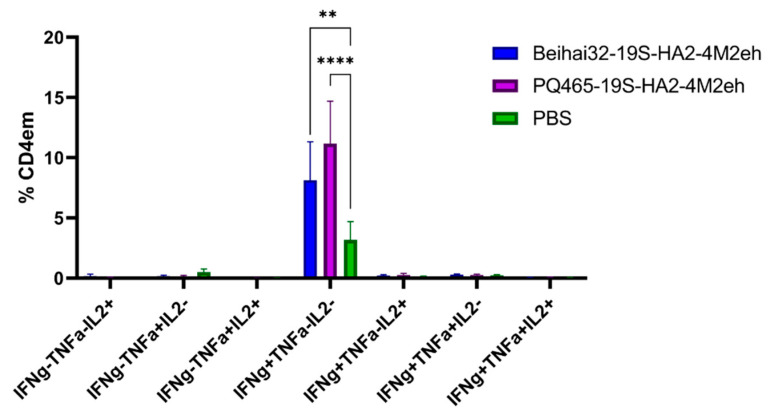
Cytokine profile of virus-specific CD4^+^ Tem in the spleens of immunized mice (5 per group). The *p* values between groups are indicated (** *p* < 0.01; **** *p* < 0.0001). Exact *p*-values are shown in Supplementary Table S1.

Immunization with chimeric Beihai32-19S-HA2-4M2eh and PQ465-19S-HA2-4M2eh particles resulted in the formation of virus-specific CD4^+^ Tem cells in the spleens of mice, while virus-specific CD8^+^ Tem cells were not detected. The post-vaccination T-cell response was characterized by the presence of virus-specific CD4^+^ Tem mono-producers of IFN-γ (8.12% in the Beihai32-19S-HA2-4M2eh group and 11.17% in the PQ465-19S-HA2-4M2eh group), with a significant difference from the control (*p* < 0.01) (Figure 13). The amounts of polyfunctional virus-specific CD4^+^ Tem cells co-expressing two or three cytokines did not differ significantly in the experimental and control groups.

**Figure 14:**
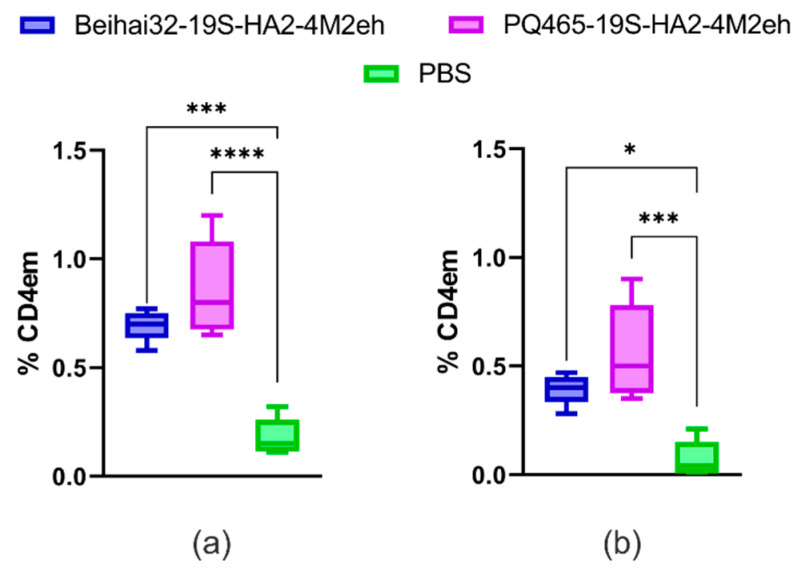
Percentage of virus-specific CD4^+^ Tem cells in spleens expressing the CD107a degranulation marker (**a**) and simultaneously CD107a and IFN-γ (**b**) after stimulation with influenza virus A/Aichi/2/68 (H3N2). Data are presented in the form of Tukey plots. The *p* values between groups (5 mice per group) are indicated (* *p* ˂ 0.05; *** *p* < 0,001; **** *p* < 0.0001). Exact *p*-values are shown in Supplementary Table S1.

The cytotoxic activity of antigen-specific CD4^+^ and CD8^+^ Tem cells was assessed by the expression of the degranulation marker (CD107a) on the surface of virus-stimulated cells and by quantitation of Tem cells simultaneously expressing the CD107a marker and producing IFN-γ. Immunization with chimeric Beihai32-19S-HA2-4M2eh and PQ465-19S-HA2-4M2eh VLPs led to an enhanced expression of the CD107a marker on the surface of virus-specific CD4^+^ Tem cells (0.69% and 0.86%, respectively) (Figure 14).

Furthermore, polyfunctional virus-specific CD4^+^ Tem expressing CD107a and producing IFN-γ were identified (0.39% and 0.56%, respectively) (Figure 14). No significant differences were observed between the experimental and control groups in the expression of the CD107a marker on virus-specific CD8^+^ Tem.

## 4. Discussion

Since current influenza vaccines induce neutralizing antibodies that target the highly variable hemagglutinin and neuraminidase proteins, developing effective vaccines that can confer protection against various influenza virus strains continues to be a substantial problem. Traditional vaccines are mainly useless in preventing future pandemics and only partially efficient in protecting people from new seasonal strains. However, by recognizing conserved viral epitopes [48], inducing effector responses to coordinate humoral and cellular immunity, and creating a population of memory cells that persist for decades, T-lymphocytes have the capacity to offer long-term cross-protection [49]. Modern broad-spectrum influenza vaccine development increasingly focuses on engineered recombinant proteins incorporating multiple conserved viral antigens (e.g., from M1, M2, HA2, NP) to achieve broad-spectrum protection [7,8].

Recent advances in the development of universal influenza vaccines based on VLPs includes the display of influenza antigens using strategies such as genetic fusion, co-expression with viral structural proteins, or protein conjugation [32]. Genetic fusion of antigens to the VLP monomer ensures highly efficient presentation, maximizing surface antigen display. However, genetic fusion of epitopes to VLPs can interfere with the assembly of chimeric particles. In these cases, the separate expression of a backbone protein and antigens within a single cell can be used, allowing them to self-assemble into VLPs [32]. The protein conjugation method, based on covalent bond formation between polypeptides [50], facilitates the separate production of VLPs and antigens in different expression systems and their subsequent ligation. Chemical conjugation allows the attachment of large, structured epitopes [51,52,53]. Influenza A virus antigens can also be linked to VLPs via isopeptide bond formation using the SpyTag-SpyCatcher platform [54]. In a recent study, influenza vaccine candidate was developed by displaying the HA stem trimer on the surface of phage AP205-based VLPs using SpyTag/SpyCatcher technology [55]. Of all these platforms, the genetic fusion method is the simplest and provides the most efficient surface antigen display if the antigen insertion does not disrupt VLP assembly.

A genetic fusion approach has previously been used to obtain VLPs based on the CPs of ssRNA phages carrying either M2e peptides [25] or HA fragments [56], but not both antigens simultaneously. In one study, VLPs based on the CP of phage AP205 carrying both M2e and the long alpha-helix from the HA stalk were constructed. In this study, 3xM2e peptide was genetically fused to the CP and expressed as a single protein, yielding stable chimeric particles (AP-M2e), while the HA-derived peptide was chemically coupled to the surface of purified AP-M2e VLPs [57]. Although AP-M2e VLPs protected mice against standard virus challenge in mice, only the combination of both HA and M2e into a single VLP fully protected mice from a high-dose homologous H1N1 influenza infection [57]. In the present work, we showed that the genetic fusion method can be successfully used to obtain chimeric VLPs based on the capsid proteins of the phages Beihai32 and PQ465, simultaneously displaying influenza-derived M2e and HA2 peptides.

Several M2e-based vaccine candidates have been developed, demonstrating induction of strong M2e-specific antibody responses and protection against influenza A challenge in animal models [7,58,59,60,61]. However, anti-M2e antibodies cannot neutralize the virus and directly prevent infection [62]. Antibodies against M2e can bind to the M2 protein expressed on infected cells and thus reduce the spread of the virus. Other protective mechanisms include M2e antibody-mediated elimination of infected cells through complement-dependent cytotoxicity (CDC), antibody-dependent cellular cytotoxicity (ADCC), and/or antibody-dependent cellular phagocytosis. Additionally, M2e-specific T cells may contribute to protection against influenza infection [63,64]. To overcome limitations of M2e-based vaccines we have added HA2 fragment of influenza A virus.

In contrast to the variable HA1 domain of hemagglutinin, the HA2 stalk region exhibits significantly higher sequence conservation. Various HA2 subdomains are currently being evaluated as potential antigens for universal influenza vaccines [19]. We used the 76–130 a.a. region of HA2, which encompasses its major α-helical domain, as this segment has demonstrated efficacy in vaccine development [18].

It should be underlined that M2e and HA2 peptides are poorly immunogenic in their native forms without adjuvants. VLPs represent a powerful platform for enhancing antigen immunogenicity, as evidenced by multiple studies [26,27,28,29,30]. The combined induction of humoral and cellular immunity underscores VLPs’ utility in modern vaccine design.

We have succeeded in the development of M2e-based vaccine candidate utilizing CPs of ssRNA phages Beihai32 and PQ465 [38]. Four copies of the M2e peptide were linked to the C-terminus of the CP of phage Beihai32 and to the N or C termini of the CP of phage PQ465. Subcutaneous administration of VLPs displaying four C-terminal M2e copies elicited robust M2e-specific IgG response in mice, conferring protection against lethal influenza challenge. In contrast, N-terminal M2e fusion to PQ465 CP yielded substantially lower immune response [38]. Thus, in the current study we have used the C-terminus of CP to link M2e and HA2 fragments. Our study confirmed that the chimeric VLPs could self-assemble in vivo, with both M2e and HA2 displayed on the particle surface. At the same time, the attached antigens reduced the carrier’s accessibility to CP-specific antibodies.

Interestingly, the sizes of Beihai32-19S-HA2-4M2eh and PQ465-19S-HA2-4M2eh particles according to TEM were close to the size of VLPs formed by empty capsid proteins of these phages (~30 nm [65]), although the fusion proteins were about twice longer. At the same time, the results of dynamic light scattering analysis revealed large sizes of chimeric VLPs. Probably the chimeric VLPs have core–shell architecture, representing a dense CP-made core and a diffuse surface layer formed by displayed antigens, not revealed by TEM.

To induce a strong immune response, booster doses of the vaccine are often necessary. A strong immune response against the carrier protein may reduce the ability of chimeric VLP to enhance the immune response against the target antigen. However, our data on the antigenic characteristics of chimeric VLPs based on CPs of phages Beihai32 and PQ465 indicated effective shielding of the particle’s CP-formed core by the antigens displayed on surface. Therefore, a strong immune response against the carrier seems unlikely, although this issue requires experimental verification. Preexisting immunity to the carrier protein in the human population may also be a problem. Although the likely hosts of phages Beihai32 and PQ465 are enterobacteria, CPs of bacteriophages of the family *Fiersviridae* have low amino acid sequence conservation, which makes the likelihood of preexisting immunity to them low.

Both chimeric VLPs induced high titers of M2e-specific antibodies in immunized mice, consistent with our previous work on CPs containing solely the M2e peptide. Since the recombinant proteins included the HA2 fragment, we also evaluated the production of influenza A/Aichi/2/68 (H3N2)-specific antibodies in serum and BAL. Immunization with Beihai32-based VLPs elicited virus-specific IgG antibodies in both serum and BAL. In contrast, PQ465-based VLPs induced virus-specific antibodies only in serum. We also evaluated IgG1 and IgG2a subclass production, which reflect Th2- and Th1-type immune responses, respectively. Interestingly, immunization with Beihai32-19S-HA2-4M2eh induced a more balanced Th1/Th2 response with an IgG1/IgG2a ratio close to 1:1, whereas a stronger Th2 response was induced in the case of PQ465-19S-HA2-4M2eh particles. Th1-type immune response plays a leading role in the control and elimination of influenza virus, although optimal protection is achieved through a balanced interplay of Th1 and Th2 responses [66,67].

T-cell immune response plays a key role in protection against influenza infection [68,69,70,71,72]. The immune response to the influenza virus depends on the interplay of cytotoxic T-cells, T helper lymphocytes, and antibody-producing B cells. Since *C57BL*/*6* (B6, H2b) mice are known to be a good model for characterization of the T-cell response to influenza infection [73], we used them to study the induction of the T cell response after immunization with VLPs. Immunization with Beihai32-19S-HA2-4M2eh particles led to the formation of M2e-specific CD4^+^ and CD8^+^ Tem responses, whose functional profile was characterized by the presence of IL-2 and TNF-α producers. In contrast, immunization with PQ465-19S-HA2-4M2eh VLPs resulted only in the formation of M2е-specific CD8^+^ Tem cells producing IL-2. It was also found that immunization with Beihai32-19S-HA2-4M2eh and PQ465-19S-HA2-4M2eh particles induced the formation of virus-specific CD4^+^ Tem cells producing IFN-γ.

CD4^+^ T cells are key participants in the formation of both humoral and cellular immune responses during influenza vaccination, contributing to the protection against infection. СD4^+^ T cells activate B cells, thereby playing a role in antibody production [74]. By secreting cytokines, CD4^+^ T cells enhance the proliferation and activity of CD8^+^ cytotoxic T lymphocytes, which are essential for eliminating infected cells [69]. In addition to their helper activity, CD4^+^ Tem cells play a protective role during influenza infection, which has been described in animal models [75,76]. CD4^+^ Tem cells provide protection through the production of cytokines, primarily IFN-γ and TNF [75], as well as through direct cytolytic activity mediated by both perforin and FAS [76].

CD8^+^ T cells not only have cytotoxic activity but also secrete chemokines that recruit other immune cells. The production of cytokines such as IFN-γ enhances their cytotoxic potential and regulates both innate and adaptive immunity, directing the immune response along the Th1 pathway. IFN-γ increases the expression of granzymes and perforin in CD8^+^ T cells, leading to the induction of apoptosis in target cells [77].

Immunization of mice with chimeric VLPs induced the formation of antigen-specific CD4^+^ and CD8^+^ Tem cells producing cytokines IL-2, TNF-α, and IFN-γ. IL-2 is a key factor in modulating the cytotoxic activity of CD4^+^ T cells. It promotes the differentiation of CD4^+^ T cells into cytotoxic cells capable of lysing infected cells. Under IL-2 influence, CD4^+^ T cells can begin to express granzyme B and perforin—key molecules involved in the induction of apoptosis in target cells [78]. TNF-α has antiviral activities and mediates the activation of phagocytic cells and inflammatory responses [79].

After immunization of mice, the emergence of virus-specific CD4^+^ Tem cells expressing the degranulation marker CD107a+ and IFN-γ was observed, suggesting that these T cells are multifunctional. There were no significant differences between the groups of mice immunized with Beihai32-19S-HA2-4M2eh and PQ465-19S-HA2-4M2eh particles. CD107a belongs to the LAMP family and represents highly glycosylated membrane proteins of lytic granules containing granzyme and perforin [80]. It has been suggested that T cells expressing CD107a and IFN-γ exhibit cytotoxic activity against the influenza virus.

Comparing the T-cell response induced by immunization with Beihai32-19S-HA2-4M2eh and PQ465-19S-HA2-4M2eh particles, it can be noted that Beihai32-19S-HA2-4M2eh more effectively stimulated the formation of M2e-specific CD4^+^ and CD8^+^ Tem cells, while virus-specific CD4^+^ Tem cells in the spleen were induced slightly more effectively in the case of PQ465-19S-HA2-4M2eh particles. These differences may be related to the better accessibility of M2e antigens on Beihai32-based VLPs. Characterization of VLP structure using more sophisticated techniques such as cryo-electron microscopy may elucidate the relationship between VLP structural features and their immunological properties. Overall, chimeric VLPs displaying conserved influenza virus antigens induced the formation of antigen-specific multifunctional CD4^+^/CD8^+^ effector memory T cells, which have the potential to provide long-term cross-protection.

It should be emphasized that we have currently characterized only the immunogenicity of the engineered VLPs. In the future, we plan to conduct a comprehensive analysis of the immune response and, most importantly, challenge studies with representative influenza A viruses from both phylogenetic groups and with antigenically distinct strains. The results of these experiments will allow us to evaluate the feasibility of developing “universal” influenza vaccines based on engineered VLPs.

## 5. Conclusions

This study demonstrated the possibility of simultaneous display of conserved influenza A antigens, the M2e peptide and the HA2 (a.a. 76–130) fragment of hemagglutinin, on VLPs formed by the capsid proteins of phages Beihai32 and PQ465. The immunization of mice with chimeric VLPs induced humoral immune response against M2e and the influenza virus, and led to the formation of cytokine-secreting antigen-specific CD4^+^ and CD8^+^ Tem cells. The observed Th1/Th2-balanced response and cross-reactive T-cell activation suggest the potential for broad protection against diverse influenza strains, although this assumption needs to be verified through challenge experiments. The engineered chimeric VLPs comprising both M2e and HA2 could be considered for future development as a universal vaccine candidate.

## Data Availability

The data presented in this study are contained within the article.

## Supplementary Materials

The following supporting information can be downloaded at: https://www.mdpi.com/article/10.3390/vaccines13111117/s1, Figure S1: Amino acid sequences of recombinant proteins; Figure S2: Analysis of VLPs formed by Beihai32-19S-HA2-4M2eh and PQ465-19S-HA2-4M2eh proteins using dynamic light scattering; Figure S3: Gating strategy for analysis of tissue-resident memory T cells; Figure S4: Representative plots of virus-specific cytokine-producing CD4^+^ Tem cells; Table S1: Statistical significance of differences between groups of animals.
