# Supplementary material for: Chimeric Virus-like Particles Formed by the Coat Proteins of Single-Stranded RNA Phages Beihai32 and PQ465, Simultaneously Displaying the M2e Peptide and the Stalk HA Peptide from Influenza a Virus, Elicit Humoral and T-Cell Immune Responses in Mice
Source: Vaccines (Basel). 2025 Oct 30;13(11):1117. doi: 10.3390/vaccines13111117 (PMC12656288; doi:10.3390/vaccines13111117)
Supplement: Supplementary file 1 [file vaccines-13-01117-s001.zip › Figure S1.pdf]

Beihai32-19S-HA2-4M2eh

MKHHHHHHPMSKPIAIFKLRELSSDSTLFTLPGHSVTLPNTLGIVSHLPTPRKGNPGTVKTMRN
LRKTILLGAGTASERAVPIVIKTETSFPVGTTEEDRAEVLKQMASFLIEEVKNNQELAYSGYVQ
DKYFIEDLVITEGS*GTSGSSGSGSGGSGSGGGG*ELRIQDLEKYVEDTKIDLWSYNAELLVALEN
QHTIDLTDSEMNKLFEKTRRQLRENADLSLLTEVETPIRNEWGSRSNDSSDDLSLLTEVETPIR
NEWGSRSNDSSDDLSLLTEVETPIRNEWGSRSNDSSDDLSLLTEVETPIRNEWGSRSNDSSDDA
LEHHHHHH

PQ465-19S-HA2-4M2eh

MKHHHHHHPMAQHNMRLQLTSGTSLTWVDPNDFRSTFRINLNVNQKVAGAVSVYNARSEVITNR
APLVVIEGCTDACSVNRENISIRTTISGSVENKAAVLAALLDHLHNLGLARDDLVAGLLPTTIQ
PVVEYTGSGS*GTSGSSGSGSGGSGSGGGG*ELRIQDLEKYVEDTKIDLWSYNAELLVALENQHTI
DLTDSEMNKLFEKTRRQLRENADLSLLTEVETPIRNEWGSRSNDSSDDLSLLTEVETPIRNEWG
SRSNDSSDDLSLLTEVETPIRNEWGSRSNDSSDDLSLLTEVETPIRNEWGSRSNDSSDDALEHH
HHHH

Figure S1. Amino acid sequences of recombinant proteins.
Capsid proteins are shown in gray, the 19S linker is shown in italics, the HA2 region is shown in purple, four tandem repeats of the M2e peptide (SLLTEVETPIRNEWGSRSNDSSD) are shown in green, and the hexahistidine tag is underlined.
