# Supplementary material for: Chimeric Virus-like Particles Formed by the Coat Proteins of Single-Stranded RNA Phages Beihai32 and PQ465, Simultaneously Displaying the M2e Peptide and the Stalk HA Peptide from Influenza a Virus, Elicit Humoral and T-Cell Immune Responses in Mice
Source: Vaccines (Basel). 2025 Oct 30;13(11):1117. doi: 10.3390/vaccines13111117 (PMC12656288; doi:10.3390/vaccines13111117)
Supplement: Supplementary file 1 [file vaccines-13-01117-s001.zip › Figure S2.pdf]

Beihai32-19S-HA2-4M2eh

Size Distribution by Volume

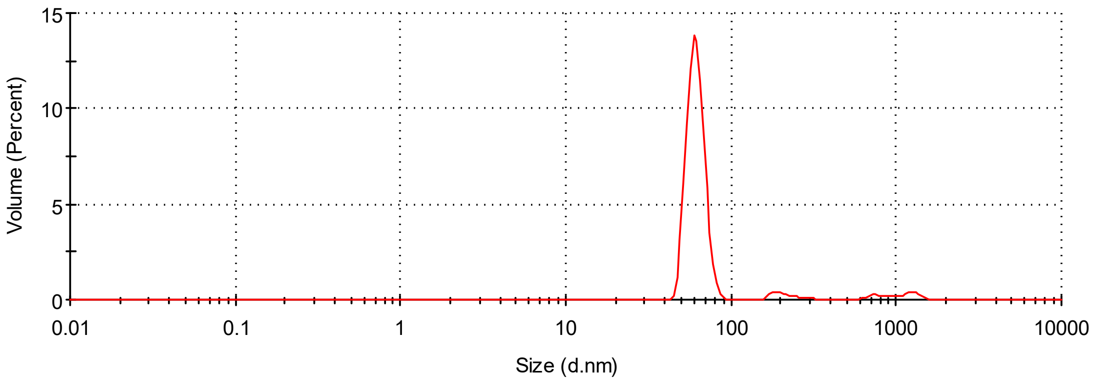

PQ465-19S-HA2-4M2eh

Size Distribution by Volume

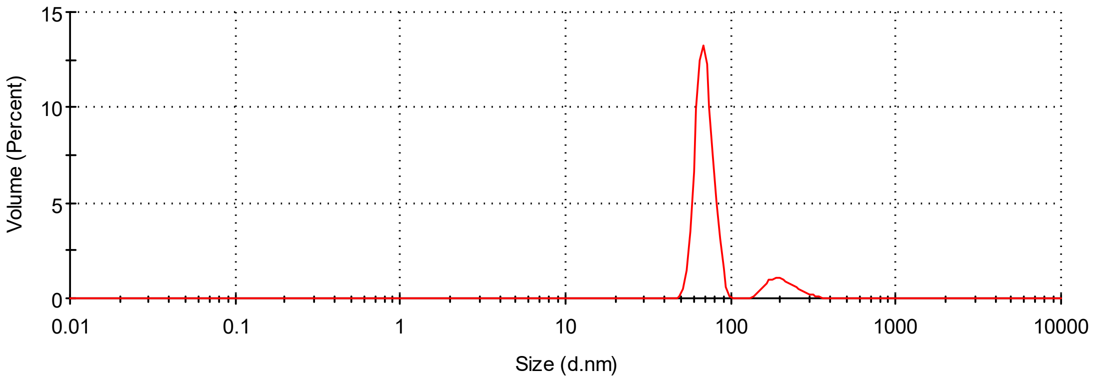

Figure S2. Analysis of VLPs formed by Beihai32-19S-HA2-4M2eh and PQ465-19S-HA2-4M2eh proteins using dynamic light scattering.

The sizes of main peaks (mean values ± standard deviation, 50 measurements):
61.4 ± 7.4 nm (Beihai32-19S-HA2-4M2eh)
69.6 ± 8.4 nm (PQ465-19S-HA2-4M2eh)
