# Supplementary material for: Chimeric Virus-like Particles Formed by the Coat Proteins of Single-Stranded RNA Phages Beihai32 and PQ465, Simultaneously Displaying the M2e Peptide and the Stalk HA Peptide from Influenza a Virus, Elicit Humoral and T-Cell Immune Responses in Mice
Source: Vaccines (Basel). 2025 Oct 30;13(11):1117. doi: 10.3390/vaccines13111117 (PMC12656288; doi:10.3390/vaccines13111117)
Supplement: Supplementary file 1 [file vaccines-13-01117-s001.zip › Figure S3.pdf]

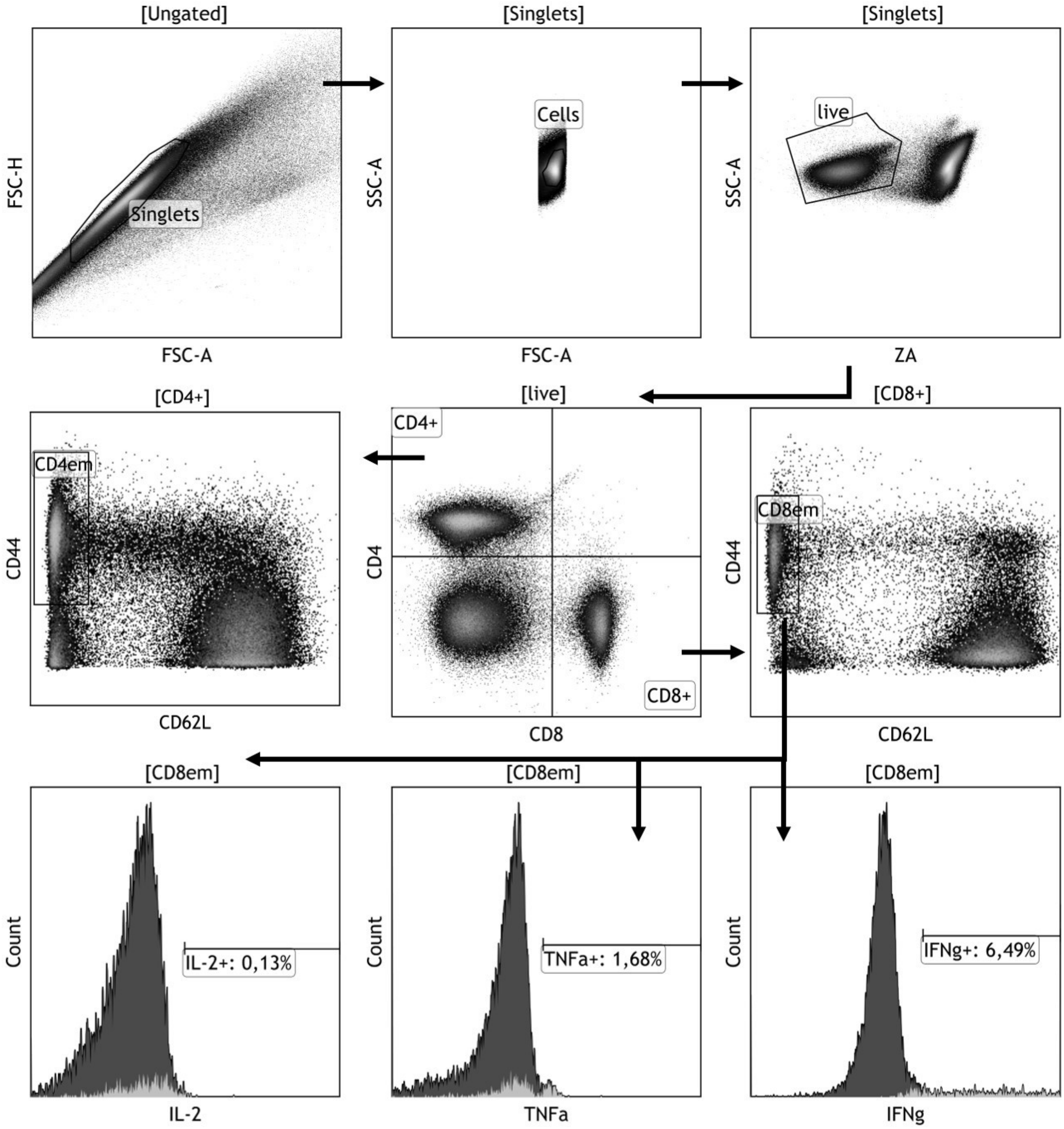

Figure S3. Gating strategy for analysis of tissue-resident memory T cells.

Non-viable cells are excluded from the analysis based on forward and side scattered light (FSC/SSC) parameters and the degree of binding of the viability marker Zombie Aqua (ZA). The population of live T lymphocytes was divided into two main subpopulations according to the presence of the surface markers CD4 and CD8: T helper ($CD4^+$) and cytotoxic T cells ($CD8^+$). Subpopulations of naive ($CD44^-$ $CD62L^+$), central (Tcm: CD44+CD62L+) and effector (Tem: $CD44^+$ $CD62L^-$) memory T cells were distinguished according to the level of expression of the CD44 and CD62L markers.
