# Supplementary material for: Chimeric Virus-like Particles Formed by the Coat Proteins of Single-Stranded RNA Phages Beihai32 and PQ465, Simultaneously Displaying the M2e Peptide and the Stalk HA Peptide from Influenza a Virus, Elicit Humoral and T-Cell Immune Responses in Mice
Source: Vaccines (Basel). 2025 Oct 30;13(11):1117. doi: 10.3390/vaccines13111117 (PMC12656288; doi:10.3390/vaccines13111117)
Supplement: Supplementary file 1 [file vaccines-13-01117-s001.zip › Figure S4.pdf]

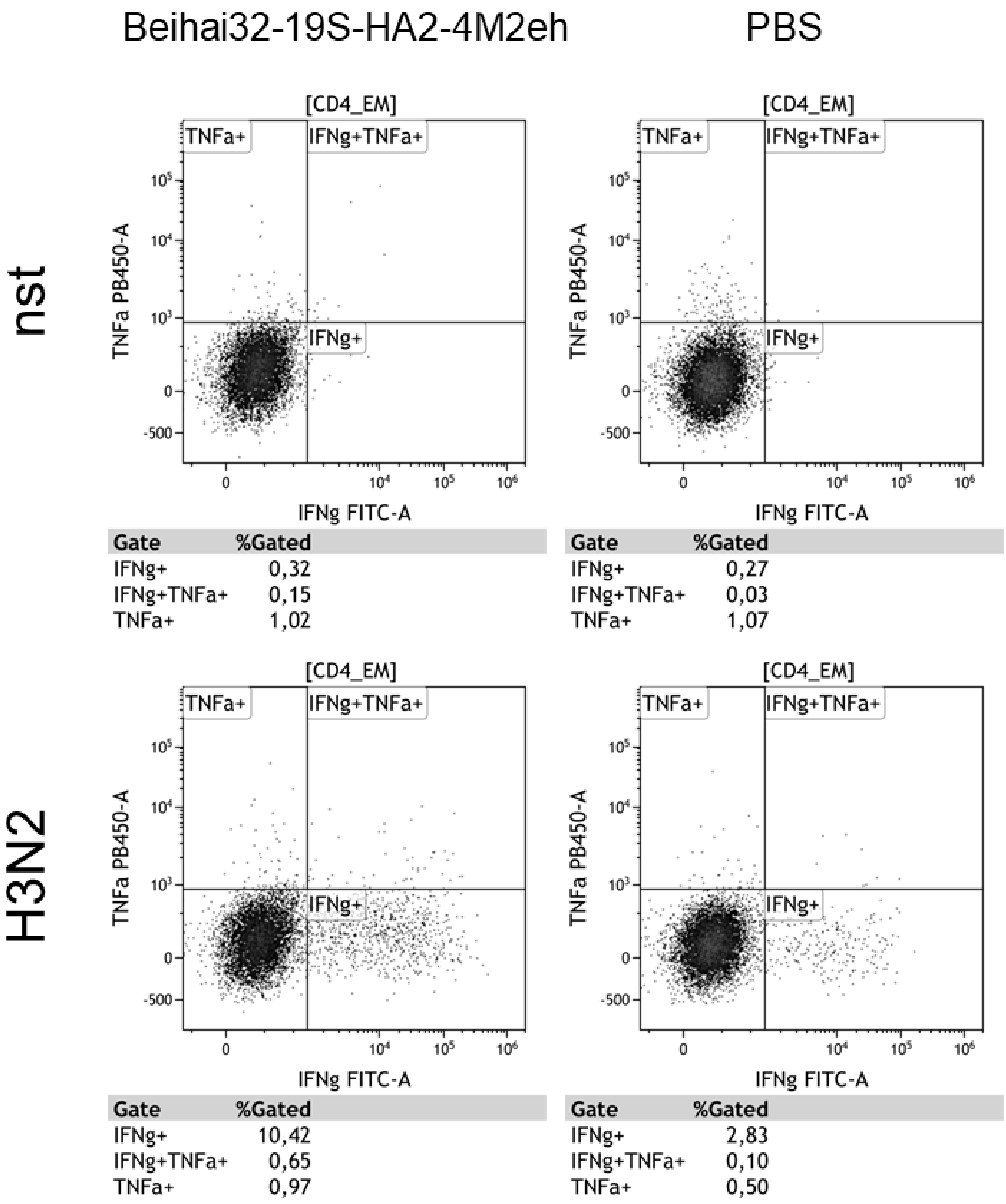

Figure S4. Representative plots of virus-specific cytokine-producing $CD4^+$ Tem cells.

Cells were isolated from spleens of mice immunized with Beihai32-19S-HA2-4M2eh particles or injected with PBS. H3N2, cells stimulated by A/Aichi/2/68 (H3N2) virus; nst, not stimulated cells.
