# Supplementary material for: Chimeric Virus-like Particles Formed by the Coat Proteins of Single-Stranded RNA Phages Beihai32 and PQ465, Simultaneously Displaying the M2e Peptide and the Stalk HA Peptide from Influenza a Virus, Elicit Humoral and T-Cell Immune Responses in Mice
Source: Vaccines (Basel). 2025 Oct 30;13(11):1117. doi: 10.3390/vaccines13111117 (PMC12656288; doi:10.3390/vaccines13111117)
Supplement: Supplementary file 1 [file vaccines-13-01117-s001.zip › Table S1.pdf]

Table S1. Statistical significance of differences between groups of animals.

Statistical significance was assessed using ANOVA in GraphPad Prism v.10.4 followed by Tukey's multiple comparisons test.

**Parameter**	**Compared groups**		***p* value**
**Figure 8(a)**			
anti-M2e IgG	Beihai32-19S-HA2-4M2eh	Beihai32	<0.0001
anti-M2e IgG	Beihai32-19S-HA2-4M2eh	PQ465-19S-HA2-4M2eh	0.53
anti-M2e IgG	Beihai32-19S-HA2-4M2eh	PQ465	<0.0001
anti-M2e IgG	Beihai32-19S-HA2-4M2eh	PBS	<0.0001
anti-M2e IgG	Beihai32	PQ465-19S-HA2-4M2eh	<0.0001
anti-M2e IgG	Beihai32	PQ465	>0.99
anti-M2e IgG	Beihai32	PBS	>0.99
anti-M2e IgG	PQ465-19S-HA2-4M2eh	PQ465	<0.0001
anti-M2e IgG	PQ465-19S-HA2-4M2eh	PBS	<0.0001
anti-M2e IgG	PQ465	PBS	>0.99
**Figure 8(b)**			
anti-M2e IgG	Beihai32-19S-HA2-4M2eh	Beihai32	<0.0001
anti-M2e IgG	Beihai32-19S-HA2-4M2eh	PQ465-19S-HA2-4M2eh	0.44
anti-M2e IgG	Beihai32-19S-HA2-4M2eh	PQ465	<0.0001
anti-M2e IgG	Beihai32-19S-HA2-4M2eh	PBS	<0.0001
anti-M2e IgG	Beihai32	PQ465-19S-HA2-4M2eh	<0.0001
anti-M2e IgG	Beihai32	PQ465	>0.99
anti-M2e IgG	Beihai32	PBS	>0.99
anti-M2e IgG	PQ465-19S-HA2-4M2eh	PQ465	<0.0001
anti-M2e IgG	PQ465-19S-HA2-4M2eh	PBS	<0.0001
anti-M2e IgG	PQ465	PBS	>0.99
**Figure 10(a)**			
virus-specific IgG	Beihai32-19S-HA2-4M2eh	Beihai32	0.039
virus-specific IgG	Beihai32-19S-HA2-4M2eh	PQ465-19S-HA2-4M2eh	>0.99
virus-specific IgG	Beihai32-19S-HA2-4M2eh	PQ465	0.17
virus-specific IgG	Beihai32-19S-HA2-4M2eh	PBS	0.017
virus-specific IgG	Beihai32	PQ465-19S-HA2-4M2eh	0.039
virus-specific IgG	Beihai32	PQ465	0.93
virus-specific IgG	Beihai32	PBS	0.99
virus-specific IgG	PQ465-19S-HA2-4M2eh	PQ465	0.17
virus-specific IgG	PQ465-19S-HA2-4M2eh	PBS	0.017
virus-specific IgG	PQ465	PBS	0.77
**Figure 10(b)**			
virus-specific IgG	Beihai32-19S-HA2-4M2eh	Beihai32	0.018
virus-specific IgG	Beihai32-19S-HA2-4M2eh	PQ465-19S-HA2-4M2eh	0.44
virus-specific IgG	Beihai32-19S-HA2-4M2eh	PQ465	0.018
virus-specific IgG	Beihai32-19S-HA2-4M2eh	PBS	0.018
virus-specific IgG	Beihai32	PQ465-19S-HA2-4M2eh	0.44
virus-specific IgG	Beihai32	PQ465	>0.99
virus-specific IgG	Beihai32	PBS	>0.99
virus-specific IgG	PQ465-19S-HA2-4M2eh	PQ465	0.44
virus-specific IgG	PQ465-19S-HA2-4M2eh	PBS	0.44
virus-specific IgG	PQ465	PBS	>0.99
**Figure 11**			

IL-2^+ cells	Beihai32-19S-HA2-4M2eh	PQ465-19S-HA2-4M2eh	0.0002
IL-2^+ cells	Beihai32-19S-HA2-4M2eh	PBS	0.0002
IL-2^+ cells	PQ465-19S-HA2-4M2eh	PBS	0.95
TNF-α^+ cells	Beihai32-19S-HA2-4M2eh	PQ465-19S-HA2-4M2eh	0.031
TNF-α^+ cells	Beihai32-19S-HA2-4M2eh	PBS	0.028
TNF-α^+ cells	PQ465-19S-HA2-4M2eh	PBS	0.96
**Figure 12**			
IL-2^+ cells	Beihai32-19S-HA2-4M2eh	PQ465-19S-HA2-4M2eh	0.55
IL-2^+ cells	Beihai32-19S-HA2-4M2eh	PBS	<0.0001
IL-2^+ cells	PQ465-19S-HA2-4M2eh	PBS	<0.0001
TNF-α^+ cells	Beihai32-19S-HA2-4M2eh	PQ465-19S-HA2-4M2eh	0.047
TNF-α^+ cells	Beihai32-19S-HA2-4M2eh	PBS	0.12
TNF-α^+ cells	PQ465-19S-HA2-4M2eh	PBS	0.67
**Figure 13**			
IFN-γ^+ cells	Beihai32-19S-HA2-4M2eh	PQ465-19S-HA2-4M2eh	0.0513
IFN-γ^+ cells	Beihai32-19S-HA2-4M2eh	PBS	0.0019
IFN-γ^+ cells	PQ465-19S-HA2-4M2eh	PBS	<0.0001
**Figure 14(a)**			
CD107a^+ cells	Beihai32-19S-HA2-4M2eh	PQ465-19S-HA2-4M2eh	0.20
CD107a^+ cells	Beihai32-19S-HA2-4M2eh	PBS	0.0003
CD107a^+ cells	PQ465-19S-HA2-4M2eh	PBS	<0.0001
**Figure 14(b)**			
CD107a^+ IFN-γ^+ cells	Beihai32-19S-HA2-4M2eh	PQ465-19S-HA2-4M2eh	0.20
CD107a^+ IFN-γ^+ cells	Beihai32-19S-HA2-4M2eh	PBS	0.010
CD107a^+ IFN-γ^+ cells	PQ465-19S-HA2-4M2eh	PBS	0.0004
